# Unexplained Findings of Kayser-Fleischer-Like Rings in a Patient with Cryptogenic Cirrhosis

**DOI:** 10.1155/2012/438525

**Published:** 2012-03-15

**Authors:** Mahreema Jawairia, Miral Subhani, Ghulam Siddiqui, Apsara Prasad, Ghulamullah Shahzad, Kaleem Rizvon, Paul Mustacchia

**Affiliations:** Department of Medicine, Nassau University Medical Center, East Meadow, NY 11554, USA

## Abstract

Cryptogenic cirrhosis (CC) is defined as cirrhosis occurring in an individual without an identifiable cause of liver disease, such as excessive alcohol consumption, viral hepatitis infection, hemochromatosis, autoimmune hepatitis, primary biliary cirrhosis, primary sclerosing cholangitis, chronic intake of medications that could induce cirrhosis, alpha-1-antitrypsin deficiency, Wilson's disease, or any other rare cause of cirrhosis according to the clinical context. Cryptogenic cirrhosis is a common cause of liver-related morbidity and mortality in the United States. Nonalcoholic fatty liver disease is now recognized as the most common cause of cryptogenic cirrhosis. A biopsy specimen is also important for detecting histological advanced disease, which may be clinically silent and undetected by liver-related tests or diagnostic imaging. We are presenting an unusual case of a patient with cryptogenic cirrhosis found to have Kayser-Fleischer-like rings without evidence of Wilson's disease.

## 1. Introduction

Cirrhosis of liver is defined as a chronic, progressive, diffuse process, characterized by fibrosis and structurally abnormal nodules in liver. Cryptogenic cirrhosis is a chronic condition of liver of unknown etiology. Cryptogenic cirrhosis accounts for 5% to 30% of cases of cirrhosis and about 10% of liver transplants [1, 2]. In the past, studies have shown that more than half of such patients are female, the average age is about 60 years, and they usually have only mild liver enzyme abnormalities. Data exist to suggest that overweight is a risk factor for chronic liver disease and that liver fibrosis can develop in overweight patients that are free of any other known causes of chronic liver disease [3].

A study by Browning et al. hypothesized that nonalcoholic steatohepatitis (NASH) associated with obesity and diabetes mellitus (DM) is responsible for the majority of cases of cryptogenic cirrhosis (CC) among Hispanics and European Americans however, their findings also indicate that this form of cirrhosis is rare among African Americans [4].

## 2. Case Presentation

**Figure 1:**
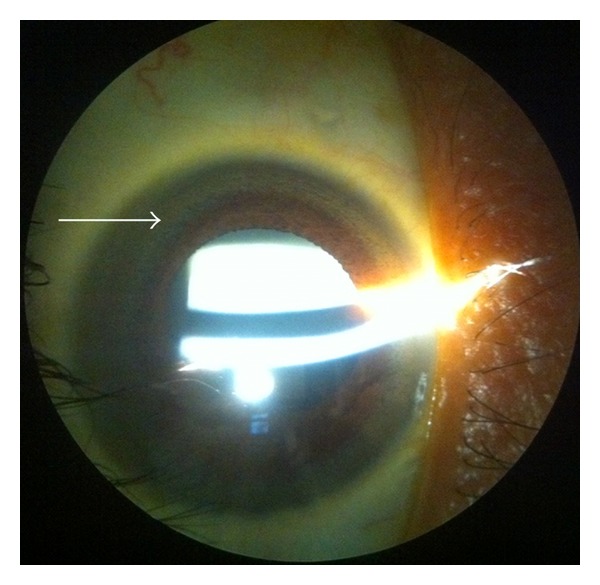
Kayser-Fleischer ring.

This is a 29 year-old Hispanic male with past medical history of cryptogenic cirrhosis diagnosed in 2002 and morbid obesity presenting with complaints of redness of left eye. Physical examination revealed anicteric sclera, conjunctivitis of left eye with a normal visual acuity of 20/20 in left eye. Abdominal examination showed distention and mild ascites. He also had bilateral edema and petechial hemorrhages in lower extremities. Laboratory tests revealed anemia with a hematocrit of 37%, severe thrombocytopenia with 44000 platelets, coagulopathy with a prolonged prothrombin time of 20.3 seconds and elevated liver tests with total bilirubin of 4 mg/dL, AST of 59 International Units, ALT of 36 International Units, and elevated alkaline phosphatase of 182. Autoimmune workup was negative, alpha-1-antitrypsin and serum ceruloplasmin levels were within normal limits and 24-hour urine copper was above the normal limit. Abdominal ultrasound showed granular and nodular appearance to the liver, compatible with the history of cirrhosis. Ophthalmology consult confirmed the presence of corneal pigmentation rings indistinguishable from Kayser-Fleischer rings on slit lamp examination (Figure 1). He was given artificial tears for dry eyes and was advised to stop wearing contact lenses. He was discharged home to followup in GI and ophthalmology clinic. When the patient followed up in GI clinic, he was scheduled for repeat liver biopsy. The CT-guided core biopsy result showed cirrhosis with 4+ iron in sinusoidal pattern and liver copper level was 179 micrograms per deciliter. Consequently, DNA testing for the C282Y and H63D mutations in the HFE gene and genetic study for ATP7B for Wilson's disease were negative.

## 3. Discussion

Cirrhosis is usually accepted as “cryptogenic” only after an extensive evaluation has omitted recognizable etiologies. Obesity and Type 2 diabetes are the most prevalent risk factors in cryptogenic cirrhosis. This suggests that many patients with cryptogenic cirrhosis represent advanced nonalcoholic steatohepatitis (NASH) [5]. Even though there is a disparity in diagnosing cryptogenic cirrhosis among pathologists working with clinicians nevertheless, epidemiologic data support NASH and silent autoimmune hepatitis as leading causes of cryptogenic cirrhosis with regional variation [6]. Liver transplantation remains the main treatment for CC. CC has been reported to account for 3% to 31% of patients with end-stage liver diseases, and it is among the most common indications for orthotropic liver transplantation [7]. Examining data from the United Network for Organ Sharing registry, Nair et al. found a stepwise increase in the diagnosis of CC with increasing BMI from 10% of overweight patients to 15% of obese patients and 18% of severely obese patients undergoing transplantation [8].

Kayser-Fleischer (K-F) rings are pigmented corneal rings at the limbus of the cornea in Descemet's membrane that have been deemed pathognomonic of Wilson's disease [9]. In 1970, Harry and Tripathi described the electron microscopic appearance of the K-F ring as electron-dense deposits of copper of varying sizes lying mainly in the Descemet's membrane [10]. According to Frommer et al., three patients, one with cryptogenic cirrhosis, one with active chronic hepatitis, and one with neonatal hepatitis, were found to have corneal pigmentation rings indistinguishable from early K-F rings on slit lamp examination. These patients did not have the clinical features of Wilson's disease and their serum copper and ceruloplasmin concentrations were normal [11]. Our patient also did not have the clinical features of Wilson's disease and his serum copper and ceruloplasmin concentrations were normal as well. While his urinary copper excretion was high, it was well below the level found in symptomatic patients with Wilson's disease. The exact nature of these rings could not be determined, and they were considered as K-F-like rings. Although K-F rings are considered as pathognomonic of Wilson's disease, they are seen in any patient with unexplained central nervous system disease, poorly categorized psychiatric disorder, abnormal liver function tests, chronic active hepatitis, cirrhosis of liver, rickets, renal tubular acidosis, and unexplained Coomb's negative hemolytic anemia [12]. Hence, it is imperative for the medical community to consider K-F rings in differential diagnoses other than Wilson's disease.
